# Use of Mendelian **R**andomization for **I**dentifying **R**isk **F**actors for **B**rain **T**umors

**DOI:** 10.3389/fgene.2018.00525

**Published:** 2018-11-12

**Authors:** Amy Elizabeth Howell, Jie Zheng, Philip C. Haycock, Alexandra McAleenan, Caroline Relton, Richard M. Martin, Kathreena M. Kurian

**Affiliations:** ^1^Brain Tumour Research Centre, Institute of Clinical Neurosciences, University of Bristol, Bristol, United Kingdom; ^2^MRC Integrative Epidemiology Unit, Population Health Sciences, Bristol Medical School, University of Bristol, Bristol, United Kingdom; ^3^Population Health Sciences, Bristol Medical School, University of Bristol, Bristol, United Kingdom

**Keywords:** Mendelian randomization, glioma, risk factors, genetic variant, causal inference, SNP, causal association

## Abstract

Gliomas are a group of primary brain tumors, the most common and aggressive subtype of which is glioblastoma. Glioblastoma has a median survival of just 15 months after diagnosis. Only previous exposure to ionizing radiation and particular inherited genetic syndromes are accepted risk factors for glioma; the vast majority of cases are thought to occur spontaneously. Previous observational studies have described associations between several risk factors and glioma, but studies are often conflicting and whether these associations reflect true casual relationships is unclear because observational studies may be susceptible to confounding, measurement error and reverse causation. Mendelian randomization (MR) is a form of instrumental variable analysis that can be used to provide supporting evidence for causal relationships between exposures (e.g., risk factors) and outcomes (e.g., disease onset). MR utilizes genetic variants, such as single nucleotide polymorphisms (SNPs), that are robustly associated with an exposure to determine whether there is a causal effect of the exposure on the outcome. MR is less susceptible to confounding, reverse causation and measurement errors as it is based on the random inheritance during conception of genetic variants that can be relatively accurately measured. In previous studies, MR has implicated a genetically predicted increase in telomere length with an increased risk of glioma, and found little evidence that obesity related factors, vitamin D or atopy are causal in glioma risk. In this review, we describe MR and its potential use to discover and validate novel risk factors, mechanistic factors, and therapeutic targets in glioma.

## The Public Health Burden of Glioma

Malignant gliomas are responsible for approximately 80% of all malignant brain tumors, with glioblastoma being the most prevalent histological subtype (Ostrom et al., 2014) (∼45% of all gliomas Ostrom et al., 2014; Visser et al., 2015). Although glioma is a relatively rare cancer, with ∼9,200 cases diagnosed each year in the United Kingdom (Cancer Research United Kingdom, 2015), the disease poses a serious health burden owing to its poor prognosis. The heterogeneous nature of the tumor cells makes the vast majority of gliomas surgically incurable (Kelly, 2010). Additionally, difficulty is faced as therapeutic agents need to penetrate the blood brain barrier (Azad et al., 2015). As a result the median survival rate of grade III gliomas is two to 5 years (Wen and Kesari, 2008) and just 15 months for glioblastoma (WHO grade IV) (Stupp et al., 2009). The 5-year survival for glioma varies, from approximately 58% for ependymoma patients to approximately 5% for glioblastoma patients (Ostrom et al., 2014; Visser et al., 2015; Cancer Research United Kingdom, 2016).

## Risk Factors for Glioma

### Accepted Risk Factors for Glioma

The only environmental factor consistently associated with glioma risk is moderate to high exposure to ionizing radiation, accounting for only a small proportion of cases (Bondy et al., 2008; Braganza et al., 2012; Urbanska et al., 2014). Evidence was first provided from the Israeli Tinea Capitus cohort of children who had undergone radiation therapy for a benign medical condition (Sadetzki et al., 2005). This was supported by data from the Childhood Cancer Survivor Study that followed-up 14,361 children and adolescents (aged < 21 at initial diagnosis) who had survived for 5 years (Neglia et al., 2006). During follow-up, 40 gliomas were diagnosed, compared to an anticipated incidence of 4.62 (standardized incidence ratios (SIR) = 8.66, 95% confidence interval (CI) 6.24–11.6). These gliomas arose at a median of 9 years after original diagnosis. In a case-control analysis (with 4 controls per case, matched on age at diagnosis, sex and time since diagnosis, and the analysis adjusted for original cancer diagnosis) the odds ratio (OR) for glioma amongst children who underwent radiation therapy vs. those who did not was 6.78 (95% CI 1.54–29.7) (Neglia et al., 2006). The authors found that the risk of glioma per Gray of radiation was greatest among children who received radiation therapy at less than 5 years of age. After adjustment for radiation dose, neither original cancer diagnosis nor chemotherapy was associated with risk (Neglia et al., 2006). Taylor et al. (2010) carried out a study of 17,980 participants who had survived at least 5 years after diagnosis of childhood cancer. In this study the risk of glioma increased linearly with dose of radiation (Taylor et al., 2010).

Rarely, glioma occurs in more than one family member, indicating a genetic susceptibility. This susceptibility is most often described within cases where inherited tumor syndromes are present, such as Li-Fraumeni syndrome, Turcot syndrome and neurofibromatosis type 1 (Louis et al., 2016). Kinnersley et al. (2018) reviewed glioma genome wide association study (GWAS) and summarized reported associations at the 27 glioma-risk SNPs (Kinnersley et al., 2018); genetic susceptibility loci are summarized in Table 1. These risk variants contribute to an increase in glioma risk; however, additional somatic mutations are a requisite for tumorigenesis in individuals with these germline variants or familial syndromes (Rice et al., 2016).

**Table 1:** Summary of the genetic susceptibility loci identified by GWAS in Europeans.

Gene	SNP	Alleles	OR (95% CI)
*TERT*	rs2736100	T/**G**	1.27 (1.19–1.37
*CCDC26*	rs4295627	**G**/T	1.36 (1.29–1.43)
*CCDC26*	rs891835	**G**/T	1.24 (1.17–1.30)
*CDKN2A/B*	rs4977756	A/**G**	1.24 (1.19–1.30)
*PHLDB1*	rs498872	C/**T**	1.18 (1.13–1.24)
*RTEL1*	rs6010620	**G**/A	1.28 (1.21–1.35)
*TP53*	rs78378222	T/**G**	2.35 (1.61–3.44)
*CCDC26*	rs55705857	A/**G**	6.3 (4.6–8.8)
Near *TERC*	rs1920116	**G**/A	1.30 (1.19–1.42)
*VTI1A*	rs11196067	**A/**T	1.19 (1.12–1.27)
*ZBTB16*	rs648044	C/**T**	1.25 (1.17–1.34)
Intergenic	rs12230172	**G**/A	1 1.23 (1.16–1.32)
*POLR3B*	rs3851634	**T**/C	1.23 (1.15–1.32)
*ETFA*	rs180159	G/**A**	1.36 (1.23–1.51)
*JAK1*	rs12752552	**T**/C	1.22 (1.15–1.31)
*MDM4*	rs4252707	G/**A**	1.19 (1.12–1.26)
*AKT3*	rs12076373	**G**/C	1.23 (1.16–1.32)
Near *IDH1*	rs7572263	**A**/G	1.20 (1.13–1.26)
*LRIG1*	rs11706832	A/**C**	1.15 (1.09–1.20)
*OBFC1*	rs11598018	**C**/A	1.14 (1.09–1.20)
Intergenic	rs11233250	**C**/T	1.24 (1.16–1.33)
*MAML2*	rs7107785	**T**/C	1.16 (1.11–1.21)
*AKAP6*	rs10131032	**G**/A	1.33 (1.22–1.44)
Near *MPG*	rs2562152	A/**T**	1.21 (1.13–1.29)
*LMF1*	rs3751667	C/**T**	1.18 (1.12–1.25)
*HEATR3*	rs10852606	T/**C**	1.18 (1.13–1.24)
*SLC16A8*	rs2235573	**G**/A	1.15 (1.10–1.20)
Near *TERC*	rs3772190	**G**/A	1.11 (1.06–1.15)
*TERT*	rs10069690	C/**T**	1.61 (1.53–1.69)
*EGFR*	rs75061358	T/**G**	1.63 (1.50–1.76)
*EGFR*	rs723527	**A**/G	1.25 (1.20–1.31)
*CCDC26*	rs55705857	**G**/A	3.39 (3.09–3.71)
*CDKN2A/B*	rs634537	T/**G**	1.37 (1.31–1.43)
*VTI1A*	rs11599775	**G**/A	1.16 (1.10–1.22)
*ZBTB16*	rs648044	**A**/G	1.19 (1.13–1.25)
*PHLDB1*	rs12803321	**G**/C	1.42 (1.35–1.49)
Intergenic	rs1275600	**T**/A	1.16 (1.10–1.21)
*RFX4*	rs12227783	**A**/T	1.16 (1.08–1.24)
*ETFA*	rs77633900	G/**C**	1.35 (1.25–1.46)
*TP53*	rs78378222	T/**G**	2.53 (2.19–2.91)
*RTEL1*	rs2297440	T/**C**	1.48 (1.40–1.56)

*Table 1 is a modified version of Table 1 in Kinnersley et al. (2018). The table describes the gene, the single nucleotide polymorphism (SNP), the allele and the odds ratio (OR) and corresponding 95% confidence interval (95% CI). ORs are reported with respect to the risk allele, highlighted in bold.*

### Other Postulated Risk Factors

There have been several risk factors that have been linked to the occurrence of glioma, though results from these investigations may be spurious because of the biases that pervade observational studies (Louis et al., 2016). A recently published systematic review presents risk factors for glioma onset that are shown to increase, decrease or have a null association with glioma risk (Quach et al., 2017).

Observational studies suggest that allergies (asthma, eczema, hay fever) are associated with lower glioma risk (Wigertz et al., 2007; Berg-Beckhoff et al., 2009; Amirian et al., 2016; Wang et al., 2016) and, consistent with this, asthma-susceptibility genotypes are associated with a reduced risk of glioma (Schwartzbaum et al., 2005). Short term use of anti-inflammatory medicine has also been reported to reduce glioma risk (Scheurer et al., 2011); although other studies have found conflicting results (Daugherty et al., 2011; Gaist et al., 2013). The possible role of allergies in decreasing the risk of glioma, including glioblastoma, may be due to an increase in immune surveillance, which in turn may destroy damaged, pro-cancerous cells earlier (Scheurer et al., 2011; Safaeian et al., 2013; Zhao et al., 2014). This hypothesis is supported by reports of a higher occurrence of glioma in HIV and AIDS patients (Blumenthal et al., 1999; Jukich et al., 2001; Hall and Short, 2009); as this is based on the result from a small number of studies with small sample sizes the estimate may be biased.

Brain tumors are observed to occur more often in Europeans compared with individuals of an African or Asian origin (McLendon et al., 1985; Kuratsu et al., 2001; Darefsky and Dubrow, 2009; Ostrom et al., 2013), an observation that has also been reported within children. Robertson et al. (2002) investigated ethnic variation in the incidence of adult brain cancer in 994,725 individuals over 10.5 years of follow-up. The authors identified 373 people who developed brain cancer (232 glioblastomas, 106 astrocytomas and 35 oligodendrogliomas) of whom 50 were of African ancestry and 323 of European ancestry. Age adjusted incidence rates (per 100,000 race specific-population/year) were 0.11 and 0.46 (*p* = 0.003) in the African and European populations, respectively. The authors report a significant difference in incidence rates for the three most common gliomas and suggest that glioma is more common in individuals of European ancestry than in individuals of African ancestry (Robertson et al., 2002). Other studies have reported that glioma occurs 3.5 times more often in Europeans compared to African Americans (Davis et al., 1999). The explanation for this observed ethnic discrepancy remains unclear and while it is possible that a genetic difference exists between the two groups (Mochizuki et al., 1999; Chen et al., 2001; Das et al., 2002), detection bias cannot be ruled out (Dubrow and Darefsky, 2011).

Certain occupations are reported to be linked with a higher risk of glioma, including physicians (Carozza et al., 2000; Krishnan et al., 2003; Pukkala et al., 2009), firefighters (Carozza et al., 2000; Krishnan et al., 2003) and farmers (Khuder et al., 1998; Zheng et al., 2001). Occupational exposure to metals such as arsenic and lead has attracted attention with respect to brain tumors as they are able to penetrate the blood brain barrier (Sunderman, 2001; Wang and Du, 2013; Liao et al., 2016). Exposure to lead has been associated with glioma risk (Anttila et al., 1996; van Wijngaarden and Dosemeci, 2006) and brain cancer mortality (Cocco et al., 1998; van Wijngaarden and Dosemeci, 2006). In a cohort study of 1,779,646 men and 1,066,346 women aged 25–64 years at baseline and subsequently followed for 19 years, an increased glioma risk was observed amongst men exposed to arsenic, mercury, and petroleum products (Navas-Acien et al., 2002). However, no relationship of lead, cadmium, nickel, chromium and iron with glioma risk was reported in a study of 1856 cases and 5189 controls (Parent et al., 2017). Other studies investigating the relationship between glioma and occupational exposure to metal (Samkange-Zeeb et al., 2010) or lead (Rajaraman et al., 2006; Bhatti et al., 2009), and between brain cancer more generally and lead (Lam et al., 2007) reported no strong evidence of a causal association.

There has been speculation that certain lifestyle choices, including alcohol intake, the use of drugs, or dietary exposure to nitrous compounds affect the risk of glioma; however, to date the evidence is inconclusive (Giles et al., 1994; Michaud et al., 2009; Kyritsis et al., 2011; Little et al., 2013; Shao et al., 2016; Tamimi and Juweid, 2017).

Mobile phone use has been speculated to be associated with brain tumor risk (Schüz et al., 2006). However, conflicting finding have also been reported (Frei et al., 2011). In a nationwide study involving Danish citizens aged 30 years or older (born after 1925), there was no evidence that mobile phone use increased brain tumor risk (Frei et al., 2011).

Other risk factors that are not discussed here have been investigated in relation to glioma risk, including but not limited to: Type 1 and type 2 diabetes, body mass index, birth weight, hypertension, height, birth weight, menarche (age at onset), menopause (age at onset), coffee/caffeine consumption, low-density lipoprotein cholesterol, insulin-like growth factor 1, insulin-like growth factor binding protein, triglycerides, high-density lipoprotein cholesterol, pesticide exposure, extremely low frequency magnetic fields, vitamin E, A and C levels (Preston-Martin and Mack, 1991; Kaplan et al., 1997; Houben et al., 2004; Linos et al., 2007; Holick et al., 2010; Kabat et al., 2011; Little et al., 2013; Malerba et al., 2013; Lee et al., 2014; Andersen et al., 2015; Li et al., 2015; Zhou et al., 2015; Seliger et al., 2016a,b; Zhao et al., 2016; Wiedmann et al., 2017).

## Observational Epidemiological Studies Vs. Mendelian Randomisation

### Problems With Observational Epidemiological Studies for Identification of Causal Risk Factors

As described above, and in common with many other diseases, the search for risk factors for glioma has largely been based on observational cohort, case-control and cross-sectional studies (Lawlor et al., 2004). Numerous cases exist of seemingly robust observational associations between putative risk factors and disease outcomes; however, interventions to modify these risk factors do not produce the anticipated benefits under randomized controlled trial (RCT) conditions (Davey Smith and Hemani, 2014). One of the postulated reasons for this is the susceptibility of observational (non-experimental) studies to several biases (specifically, confounding, measurement error and reverse causation) that can generate spurious associations and which can be difficult to eradicate even through statistical adjustment (Davey Smith and Hemani, 2014).

A confounder is a factor that is a common cause of both the disease under consideration and the exposure of interest. Importantly, a confounder is not on the causal pathway between the exposure and outcome (Hammer et al., 2009). For instance, in 2002 an association had been established between alcohol intake and the incidence of 3.6% of all cancers (Boffetta et al., 2006; Testino, 2011) but it is still uncertain whether an association exists between any class of glioma and alcohol intake (Braganza et al., 2014; Qi et al., 2014). An observed association between glioma incidence and alcohol intake could be because individuals who consume more alcohol are more likely to smoke (Hart et al., 2010) and to adhere to an unhealthy life-style; (Sayon-Orea et al., 2011; Bendsen et al., 2013) thus, it could be these other factors that influence the risk of glioma rather than alcohol consumption *per se* (Sergentanis et al., 2015).

Reverse causation occurs when the disease outcome precedes, and leads to, the exposure rather than being a consequence of the exposure (Flegal et al., 2011). For example a higher level of blood glucose has been reported to be protective against glioma (Kitahara et al., 2014); however, an alternative explanation is that tumors take-up glucose, leading to low glucose levels (Schwartzbaum et al., 2017).

### Mendelian Randomization Analogous to Randomized Control Trials (Figure 1)

**FIGURE 1:**
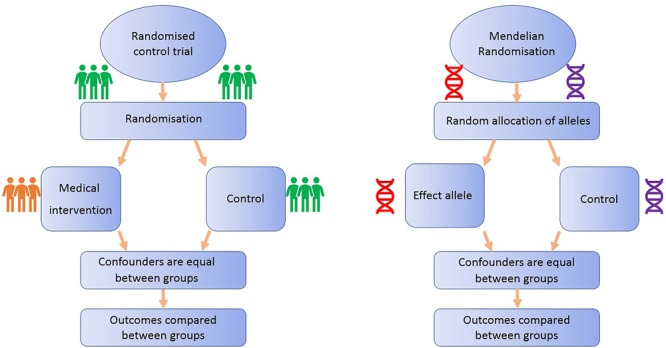
Comparison of Mendelian randomization (MR) with randomized control trial. This demonstrates the analogy between a randomized control trial and a Mendelian randomized study.

Randomized controlled trials are considered the gold standard study design for inferring causality, as successful randomization, adequately blinded implementation of the intervention, high rates of follow-up and intention-to-treat analysis should yield results that are relatively free from the biases afflicting observational studies (Iturrieta-Zuazo and Walter, 2015). On the other hand, RCTs often reflect short-term exposures at one time point in life, with limited follow-up, and participants are usually not representative of general populations, a particularly important issue if the priority is to identify primary prevention targets (Meyer, 2010). Additionally, due to ethical, practical and financial reasons, it is not feasible to randomize people to every risk factor (Bochud and Rousson, 2010): e.g., exposure to power lines, mobile phone use or breastfeeding.

One method to appraise causality within observational epidemiology is the use of Mendelian randomization (MR). MR is a type of “instrumental variable” analysis that utilizes genetic variants, such as SNPs, that are robustly associated with an exposure as proxies for the risk factor of interest. The aim of MR is to strengthen causal inference in observational studies of associations between risk factors and disease (Lawlor et al., 2008).

All MR studies make use of germline genetic data as opposed to somatic data. Germline genetic variants tend to be randomly distributed with respect to most human traits in the general population. This is because of Mendel’s laws of inheritance (segregation, independent assortment) and the fixed nature of germline genotypes (Castle, 1903). Thus, germline genetic variants are less likely to be affected by the sorts of confounding factors that typically bias observational findings (Qi, 2009). Additionally, as germline genotype cannot be affected by the presence of disease, the generation of spurious results through reverse causation is avoided (Larsson et al., 2017). Germline genetic variants can thus be regarded as randomized proxies for an exposure of interest, in the same way that the allocation group in an RCT is a proxy for an intervention of interest (Figure 1). MR can exploit SNPs that are associated with modifiable risk factors to strengthen causal inference about the nature of relationships between risk factors and disease (Larsson et al., 2017).

**FIGURE 2:**
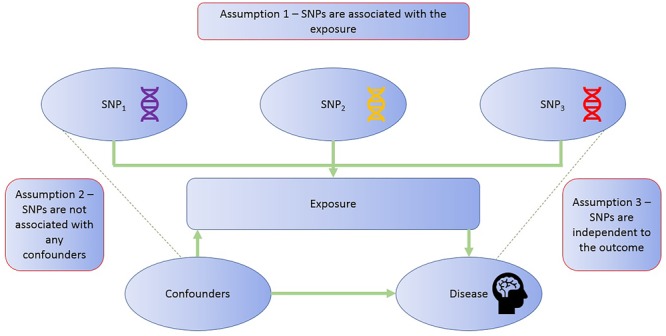
MR assumptions. The diagram illustrates the three assumptions of the MR methodology.

The application of MR involves three assumptions (Figure 2): (1) the genetic variants (“instruments”) are reliably associated with the risk factor of interest; (2) the genetic variants are independent of confounding factors (Didelez and Sheehan, 2007; VanderWeele et al., 2014); and (3) the genetic variants are only associated with the disease outcome through the risk factor of interest (Greenland, 2000; Lawlor et al., 2008). Within the constraints of these assumptions, genetic instruments (SNPs) can be used as proxies for a large range of cancer-related modifiable exposures. One-sample MR is the standard application of MR. There is one data set that contains all the data on the SNPs, exposure, and outcome for all participants (Haycock et al., 2016). Due to the rare nature of glioma, one-sample MR is likely to be statistically underpowered. As a result, MR techniques have been developed to allow analysis when genetic association studies are conducted in two separate samples sets: one set for the exposure of interest and one for the outcome (Inoue and Solon, 2010). This method is referred to as **two-sample MR** (Hartwig et al., 2016).

Like most diseases, glioma GWAS to date have examined genetic variation in relation to the causes of disease risk, using case-control study designs, as opposed to disease progression (Melin et al., 2017). The primary application of MR in glioma research has, therefore, focused primarily on causal effects of environmental exposures on disease risk (Walsh et al., 2015; Haycock et al., 2017; Disney-Hogg et al., 2018a; Takahashi et al., 2018), as opposed to survival. There are some instances where factors are involved in both disease incidence and progression, such as low-density lipoprotein cholesterol levels for heart disease risk and recurrence (Ference et al., 2017), although such instances may be exceptional. Cases do exist where a risk factor for a disease is not implicated in progression, as has been proposed for the relationship between folate consumption and colon cancer (Kim, 2003). Thus, current case-control GWAS of glioma risk have the potential to inform on the underlying causal mechanisms of disease onset but (at the current time) may be less informative for discovering drug targets to improve glioma survival (Paternoster et al., 2017). The latter requires case-only GWAS that examine genetic variation in relation to disease progression, but such studies are currently rare (Melin et al., 2017). The most probable explanation for this is due to a research focus to determine mechanisms that cause disease incidence and because of the challenges inherent in collecting progression data (see section “Future of MR in research” below). At present, a few MR studies have been conducted that investigate progression of disease (Brunner et al., 2017) but none in glioma progression, which is required for the discovery of targets for improving glioma survival (Paternoster et al., 2017).

Mendelian randomization can be used to identify and investigate potential drug targets (Mokry et al., 2015; Zheng et al., 2017). A quarter of the drugs that enter clinical development fail due to their ineffectiveness (Ashburn and Thor, 2004; Arrowsmith and Miller, 2013). Current drug targets are authenticated using *in-vitro* and animal models, but these can fail to predict the potential benefits (or harms) in humans (Mokry et al., 2015; Zheng et al., 2017). Nelson et al. (2015) aimed to establish whether current genetic evidence could predict drug mechanisms. The authors reported that opting for targets that are genetically supported may result in twice the success rate in clinical development (Nelson et al., 2015). MR could substantially augment these methods (Mokry et al., 2015; Zheng et al., 2017). The theory is that specific genetic variants can be utilized to imitate the effects of targeting a protein pharmacologically. If the variant codes for a potential drug target that causes an alteration in activity of the encoded protein, the causal effect of the drug on disease can be assessed by MR (Sofat et al., 2010; Evans and Smith, 2015). Additionally, MR can be used to examine all pairwise associations between serum protein levels and disease risk (Sun et al., 2018). If a variant is identified that is robustly associated with levels of a serum protein that display a putative causal relationship with disease risk, methods can be employed to search for available drugs that cause an alteration in the levels of that protein (Corsello et al., 2017). As discussed, only case-control GWAS exist at present for glioma which may be less informative for the discovery of drug targets to improve survival (Paternoster et al., 2017).

Table 2 provides a summary of some of the different methods used to obtain MR estimates (Hemani et al., 2018).

**Table 2:** Description of statistical methods used in Mendelian randomization analysis.

Statistical Method	Description
Inverse-variance weighted (IVW)	Assumes causal estimate due to each SNP is the same (fixed effects IVW) or that if their effects differ that their deviations are balanced (random effects IVW) (Hemani et al., 2018). Gives an unbiased estimate when there is no horizontal pleiotropy (fixed effects IVW) or when horizontal pleiotropy is balanced (random effects IVW).
Maximum likelihood estimation (MLE)	Assumes effect of the exposure on the outcome due to each SNP is equal (fixed effects IVW makes the same assumption). A benefit of this method is that it might give more reliable results when measurement error in the SNP-exposure effect is present (Hemani et al., 2018). Gives an unbiased estimate when there is no horizontal pleiotropy or when horizontal pleiotropy is balanced (but variance of the estimate will be underestimated in the latter scenario).
Weighted median estimate (WME)	Takes the median effect of all SNPs. Returns an unbiased estimate if half the SNPs are valid instruments (Hemani et al., 2018). Requires a large number of instrumental SNPs otherwise method is underpowered.
Mode-based estimate (MBE)	SNPs are clustered into groups determined by similarity of causal effects. Returns the causal effect estimate based on the cluster that has the greatest number of SNPs (Hemani et al., 2018). Gives an unbiased estimate if the SNPs in the largest cluster are valid, even if most SNPs are invalid instruments. Requires a large number of instrumental SNPs otherwise method is underpowered.
MR-Egger	Modifies the IVW analysis by permitting a non-zero intercept, permitting the net-horizontal pleiotropic effect for all SNPs to be unbalanced, or directional (Hemani et al., 2018). Gives an unbiased estimate even if all SNPs do not adhere to instrumental variable assumptions but requires the InSIDE (instrument strength independent of direct effects) assumption to be valid. Requires a large number of instrumental SNPs otherwise method is underpowered.
Wald ratio	This is the easiest method to estimate a causal effect. Wald ratio method is appropriate when only a single SNP is available to proxy the risk factor of interest. However, a limitation is that it is much harder to appraise MR assumptions with only a single SNP. (Wald, 1940).

*The statistical methods described are the inverse variance weighted (IVW), maximum likelihood estimation (MLE), weighted median estimate (WME), mode-based estimate (MBE) and MR-Egger.*

### Limitations of MR Pertinent to Glioma

Mendelian randomization has widely recognized limitations (Glynn, 2010). For some exposures there is a lack of genetic variants (SNPs) available for instrumentation (Smith and Ebrahim, 2004). For example, ionizing radiation emitted by mobile phones has been suggested as a risk factor for glioma (Yang et al., 2017). However, currently no genetic variants have been associated with exposure (or response) to ionizing radiation and therefore MR analysis cannot be performed for this particular risk factor (Smith, 2010).

A key limitation of MR is pleiotropy (Sheehan et al., 2008). Pleiotropy occurs when a genetic variant has more than one effect. If one or more of these effects influence the outcome through pathways other than the exposure of interest (so called horizontal pleiotropy) a core MR assumption is violated, i.e., that variants only exert their effect on the outcome via their influence on the exposure of interest (Evans et al., 2013; Burgess, 2014; Bennett and Holmes, 2017; Yarmolinsky et al., 2017). Techniques have been developed, such as MR-Egger regression, that can quantify the amount of bias caused by horizontal pleiotropy, as well as providing a valid causal estimate despite the presence of horizontal pleiotropy (Bowden et al., 2015). Another type of pleiotropy that exists is vertical pleiotropy. This is where the genetic variants have associations with biomarkers that are downstream of the biomarker of interest (Bennett and Holmes, 2017). Thus, they are on the causal pathway and should be considered as intermediates of the relationship between an exposure and an outcome, not as confounding factors.

Mendelian randomization studies typically require large sample sizes, an issue that can be compounded by the rare nature of glioma. One way to increase power is to develop genetic risk scores that contain multiple alleles to explain more of the variance in the exposure of interest. This runs the risk of including invalid variants, such as those that do not exert their effect on the outcome via the exposure of interest (Evans et al., 2013; Burgess, 2014; Yarmolinsky et al., 2017), although such potential violations of the MR assumption can be formally tested using MR-Egger regression. Power can also be increased by using a two-sample approach, where large case-control GWAS can be used even if they have not measured the exposure of interest.

Limitations of MR have been discussed in detail in several published papers (Davey Smith and Ebrahim, 2003; Smith and Ebrahim, 2004; Lawlor et al., 2008; Sheehan et al., 2008; Bochud and Rousson, 2010; Davey Smith, 2011b; VanderWeele et al., 2014).

## Mr in Glioma Research

### Studies That Have Evaluated Risk Factors for Glioma Using MR

Two-sample MR is a method that can harness information from GWAS summary statistics and has been applied to the context of glioma to look at several risk factors. We discuss key studies that have used two-sample MR to investigate associations between previously reported risk factors and glioma (Table 3).

**Table 3:** Description of MR studies that have investigated the causal association between a factor and glioma risk.

Author of the study	Number of glioma	Risk factor of interest	Main Finding
	cases and controls		
Haycock et al., 2017	1,130 cases and 6,294 controls	Telomere Length	Risk of glioma increases per standard deviation (SD) increase in telomere (OR 5.27; 95% CI: 3.15–8.81. *P* = 0.01)
Walsh et al., 2015	1,130 cases and 6,294 controls	Telomere Length	Risk of glioma increases monotonically with each increasing septile of telomere length (O.R 1.12; 95% CI: 1.09–1.16. *P* = 3.83 × 10^-12^)
Takahashi et al., 2018	12,488 cases and 18,169 controls	Vitamin D levels	Little evidence of any association. (OR per SD increase in Vitamin D levels 1.21; 95% CI: 0.90–1.62. *P* = 0.201)
Disney-Hogg et al., 2018a	12,488 cases and 18,169 controls	Atopy	For binary risk factors the results can be interpreted by risk of disease/odds ratio for glioma per 2.7-fold increase in odds of the risk factor (exposure). No strong evidence of any association between glioma and asthma and hay fever (OR 0.96; 95% CI: 0.90–1.03. *P* = 0.248), IgE levels (OR 0.88; 0.69–1.13. *P* = 0.319), or self-reported allergy (OR 1.03; 95% CI: 0.95–1.11. *P* = 0.534). For atopic dermatitis an inverse association was found by IVW (OR 0.96; 95% CI: 0.93–1.00. *P* = 0.041) and MLE (OR 0.96; 95% CI: 0.94–0.99. *P* = 0.003)
Disney-Hogg et al., 2018b	12,488 cases and 18,169 controls	Obesity-related factors	No strong evidence of any association for all factors (*P* = > 0.05).

An MR study to evaluate the causal relevance of telomere length on the risk of cancer and non-neoplastic diseases found that genetically predicted longer telomeres increased the risk of glioma, while being protective for certain non-neoplastic diseases, such as cardiovascular diseases (Zheng et al., 2017). The analysis employed summary genetic data for 35 cancers and 45 non-neoplastic diseases, including 1,130 glioma cases and 6,294 controls. The strongest association was for glioma (OR per SD increase in genetically predicted telomere length was 5.27; 95% CI: 3.15–8.81) (Zheng et al., 2017). A possible explanation for this observation is that telomere shortening may act as a tumor suppressor, restricting the proliferative potential of cells. Therefore, those with longer telomeres have a greater probability of obtaining somatic mutations due to an increased proliferative potential (Hanahan and Weinberg, 2011).

Walsh et al. (2015) also used an MR approach to establish whether a genotypically estimated longer or shorter telomere length was linked with an increased risk of glioma and whether inheritance of SNPs associated with telomere length are indicators of glioma risk. The authors accessed differences in genotypically estimated relative telomere length in a total of 1,130 glioma patients and 6,294 controls. The average approximated telomere length was 31bp (5.7%) longer in glioma cases compared with controls in discovery analyses (*P* = 7.82 × 10^-8^). This finding was supported in the replication analysis as the mean telomere length was 27 bp (5.0%) longer in glioma cases than controls (1.48 × 10^-3^). The authors reported that the risk of glioma increases monotonically with each increasing septile of telomere length (O.R 1.12; 95% CI: 0.90–1.62). Additionally, the authors reported that four telomere length-associated SNPs were significantly related with glioma risk in pooled analyses, including those in the telomerase component genes *TERC* (O.R 1.14; 95% C.I. = 1.03–1.28) and *TERT* (O.R 1.39; 95% C.I. = 1.27–1.52), and those in the CST complex genes *OBFC1* (O.R 1.18; 95% C.I. = 1.05–1.33) and *CTC1* (O.R 1.14; 95% C.I. = 1.02–1.28). The indication of risk alleles for glioma close to *TERC* and *TERT* that are also related with telomere length suggests that telomerase is important in glioma formation (Walsh et al., 2014).

Takahashi et al. (2018) used two-sample MR to investigate whether a causal relationship exists between circulating vitamin D and glioma risk, involving 12,488 glioma cases and 18,169 controls. The authors reported no strong evidence of a causal relationship between vitamin D and glioma when either the inverse-variance weighted (IVW) method (OR per SD increase 1.21, 95% CI: 0.90–1.62, *P* = 0.201) or the maximum likelihood estimation (MLE) method (OR per SD increase 1.20, 95% CI: 0.98–1.48, *P* = 0.083) was used (Takahashi et al., 2018).

Disney-Hogg et al. (2018a) used an MR approach to evaluate the observed inverse relationship between allergies and glioma risk. The instrumental variables were SNPs robustly associated with atopic dermatitis, asthma and hay fever, IgE levels, and self-reported allergy. The study involved 12,488 cases and 18,169 controls. The authors found no significant association between glioma and asthma, hay fever, IgE levels, or self-reported allergy. For atopic dermatitis an inverse association was found (OR per 2.7-fold increase in odds of atopic dermatitis) by the IVW (OR 0.96, 95% CI 0.93–1.00, *P* = 0.041) and MLE methods (OR 0.96, 95% CI 0.94–0.99, *P* = 0.003), but not for weighted median estimate (WME) and mode-based estimate (MBE) methods (Disney-Hogg et al., 2018a), suggesting that having atopic dermatitis reduces the risk of glioma.

Disney-Hogg et al. (2018b) carried out an MR analysis to interrogate the observed association between obesity-related factors and risk of glioma. The authors identified variants that were robustly associated with 10 key obesity-related factors: 2-h post-challenge glucose, BMI, fasting glucose, fasting insulin, HDL cholesterol, LDL cholesterol, type-2 diabetes, total cholesterol, triglycerides and waist-hip ratio. This study encompassed 12,488 cases and 18,169 controls. This study found little evidence that indicated that obesity-related factors contribute to glioma (Disney-Hogg et al., 2018b).

### Potential Application of Different MR Study Designs in Glioma Research

There are several different design strategies for MR that have been discussed in detail by Zheng et al. (2017). The potential application of these different MR study designs in glioma research are outlined below.

Improved knowledge of signaling pathways that are causally associated glioma incidence can be helpful to design preventative strategies and effective therapeutic targets (Wang et al., 2015). A useful MR strategy to establish whether a molecular intermediate plays a role in the causal pathway between a risk factor and disease is the use of **two-step MR** (Relton and Davey Smith, 2012). An improved understanding of the molecular changes that drive glioma formation will allow for opportunities to modify disease causing factors.

**Bidirectional MR** involves using instruments for both the exposure and the outcome to assess the direction of causality: i.e., does the exposure cause the outcome or does the outcome cause the exposure (Timpson et al., 2010). For instance, observational studies have suggested that there is an inverse association between allergies and glioma risk, but the direction and causality of the association remains uncertain: it is not clear whether allergies decrease the risk of glioma or whether the inverse association arises because of suppression of the immune system by glioma itself (Schoemaker et al., 2006).

There are cases in which genetic variants are related to numerous correlated phenotypes (Low, 2001), for example, genetic variants that associate with lipoprotein metabolism tend not to correlate with just one specific lipid fraction (Wurtz et al., 2013). As a result assessing the causal association of one specific intermediate phenotype with disease can be challenging (Davey Smith and Hemani, 2014). **Multi-phenotype MR** can be used in these cases (Davey Smith and Hemani, 2014; Burgess et al., 2015; Burgess and Thompson, 2015; Kemp et al., 2016). Multivariable MR can be applied to glioma research when testing the effect of lipids on glioma to identify the independent effect of each lipid subtypes on glioma.

Hypothesis-driven MR has huge potential in glioma research. Hypothesis-driven MR can validate the relationship between a risk factor and glioma for which a causal association has previously been reported.

In addition, hypothesis-free MR has the potential to identify novel causal associations. Hypothesis-free MR can be used to examine causality in complex frameworks in glioma, as a well as a method to data mine high-dimensional studies (Evans and Davey Smith, 2015). Haycock et al. (2017) implemented a mixture of hypothesis-driven and hypothesis-free MR to investigate the relationship between telomere length and 22 cancers and 32 primary non-neoplastic diseases.

Mendelian randomization-Base is a tool that improves the accessibility of GWAS summary data for MR research (Hemani et al., 2016). MR-Base can assist hypothesis-free testing as it allows researchers to examine all pairwise associations to data mine for causal relationships of interest (Davey Smith, 2011a). Where novel associations are identified, these associations can then be subjected to formal and extensive hypothesis-testing studies (Evans and Smith, 2015).

**Factorial MR** can be used to develop therapeutic strategies to improve glioma survival. Factorial RCT is where a participant is either assigned to a group that obtains neither intervention, one of the interventions, or both (Montgomery et al., 2003). In a factorial trial the separate effects of each intervention can be considered, as well as, the benefits of obtaining both interventions together (Montgomery et al., 2003). Similarly, factorial MR can be performed by using combinations of genetic variants to attain unconfounded estimates of the effect of co-occurrence of the two drug targets on disease (Davey Smith and Hemani, 2014). In glioma research if we have two drug targets and we want to know the combined effects of these two drugs on glioma, then we can apply factorial MR. Factorial MR can assess the antitumor efficacy of drug targets on glioma by investigating the combination of different targeted drugs (Reardon and Wen, 2006).

### Future of MR in Glioma Research

For GWAS and MR of glioma progression to be successful for the development of drug targets to improve glioma survival, large scale case-only studies will be required with both progression and germline genetic data. RCTs offer a potential reservoir of data for such studies; however, due to the rare nature of glioma, sample size is limited (Vuorinen et al., 2003; Gehring et al., 2009; U.S.National Library of Medicine, 2018). A limitation of progression studies is the introduction of collider bias, discussed in detail in Paternoster et al. (2017). Collider bias is problematic in MR of disease progression as a risk factor of interest that causes the disease may be correlated with other risk factors involved in incidence, and any association between the index risk factor and progression can be confounded by these correlated risk factors. If the problems of sample size and collider bias can be adequately overcome, GWAS and MR of disease progression offer a promising opportunity to identify new treatments for glioma that could enhance survival (Davey Smith et al., 2017). Additionally, an improved understanding of the molecular changes that drive glioma progression will allow for opportunities to develop targeted molecular therapies. At present, although there are some examples where targeted therapy responses have been recorded in glioma patients, no targeted therapy has been approved as an effective treatment in clinical trials (Touat et al., 2017).

Future research will involve hypothesis free MR, which make use of omics data. There is a growing body of evidence showing that epigenetic biomarkers of glioma can be used for prediction and prognosis. Notably in neuro-oncology the O^6^-methylguanine-DNA methyltransferase promoter methylation can act both a prognostic and predictive biomarker for glioblastoma (Esteller et al., 2000; Olson et al., 2011; Reifenberger et al., 2012; Wick et al., 2012). As genetic variants associated with DNA methylation seem to overlap with expression quantitative trait loci (eQTLs) at many loci throughout the genome (Bell et al., 2012; Shi et al., 2014), both DNA methylation and gene expression may exist on the causal pathway between genetic variation and disease. The ability to identify epigenetic and transcriptomic markers for glioma risk and progression could be important in understanding the underlying mechanisms of glioma. Using an MR approach, the causal chain between DNA methylation, gene expression and glioma onset/progression can be investigated (Relton and Davey Smith, 2012).

Given the lack of large-scale case-only studies with data on progression and germline genetic data, a priority of research in the near term should be to identify causes of glioma onset. The findings from such studies will be informative for the design of primary and secondary prevention strategies. The latter could be particularly valuable for glioma prevention in high risk populations, such as childhood cancer survivors (who received radiation therapy), people with genetic syndromes known to increase risk of glioma and people exposed to known causal factors because of their occupations. For example, if a specific dietary factor is found to be causally associated with a decrease in glioma risk, high risk populations could be advised to increase their consumption of that specific dietary factor.

## Conclusion

Mendelian randomization offers a promising, novel way to identify risk factors and drug targets for glioma to both inform public health policy for prevention, as well as, allowing the development of therapeutic approaches to improve prognosis. The latter will require the development of large-scale case-only studies with data on progression and germline genetic data.

## Author Contributions

AH contributed to the manuscript research and writing. KK, PH, RM, CR, JZ, and AM reviewed and revised the manuscript.

## Conflict of Interest Statement

The authors declare that the research was conducted in the absence of any commercial or financial relationships that could be construed as a potential conflict of interest.

## References

[B1] Amirian E. S. Zhou R. Wrensch M. R. Olson S. H. Scheurer M. E. Il’yasova D. (2016). Approaching a scientific consensus on the association between allergies and Glioma risk: a report from the glioma international case-control study. *Cancer Epidemiol. Biomark. Prev.* 25 282–290. 10.1158/1055-9965.epi-15-0847 26908595 PMC4874516

[B2] Andersen L. Friis S. Hallas J. Ravn P. Kristensen B. W. Gaist D. (2015). Hormonal contraceptive use and risk of glioma among younger women: a nationwide case-control study. *Br. J. Clin. Pharmacol.* 79 677–684. 10.1111/bcp.12535 25345919 PMC4386952

[B3] Anttila A. Heikkila P. Nykyri E. Kauppinen T. Pukkala E. Hernberg S. (1996). Risk of nervous system cancer among workers exposed to lead. *J. Occup. Environ. Med.* 38 131–136. 10.1097/00043764-199602000-00010 8673517

[B4] Arrowsmith J. Miller P. (2013). Trial watch: phase II and phase III attrition rates 2011-2012. *Nat. Rev. Drug Discov.* 12:569. 10.1038/nrd4090 23903212

[B5] Ashburn T. T. Thor K. B. (2004). Drug repositioning: identifying and developing new uses for existing drugs. *Nat. Rev. Drug Discov.* 3 673–683. 10.1038/nrd1468 15286734

[B6] Azad T. D. Pan J. Connolly I. D. Remington A. Wilson C. M. Grant G. A. (2015). Therapeutic strategies to improve drug delivery across the blood-brain barrier. *Neurosurg. Focus* 38:E9. 10.3171/2014.12.Focus14758 25727231 PMC4493051

[B7] Bell J. T. Tsai P. C. Yang T. P. Pidsley R. Nisbet J. Glass D. (2012). Epigenome-wide scans identify differentially methylated regions for age and age-related phenotypes in a healthy ageing population. *PLoS Genet.* 8:e1002629. 10.1371/journal.pgen.1002629 22532803 PMC3330116

[B8] Bendsen N. T. Christensen R. Bartels E. M. Kok F. J. Sierksma A. Raben A. (2013). Is beer consumption related to measures of abdominal and general obesity? A systematic review and meta-analysis. *Nutr. Rev.* 71 67–87. 10.1111/j.1753-4887.2012.00548.x 23356635

[B9] Bennett D. A. Holmes M. V. (2017). Mendelian randomisation in cardiovascular research: an introduction for clinicians. *Heart* 103 1400–1407. 10.1136/heartjnl-2016-310605 28596306 PMC5574403

[B10] Berg-Beckhoff G. Schuz J. Blettner M. Munster E. Schlaefer K. Wahrendorf J. (2009). History of allergic disease and epilepsy and risk of glioma and meningioma (INTERPHONE study group, Germany). *Eur. J. Epidemiol.* 24 433–440. 10.1007/s10654-009-9355-6 19484497

[B11] Bhatti P. Stewart P. A. Hutchinson A. Rothman N. Linet M. S. Inskip P. D. (2009). Lead exposure, polymorphisms in genes related to oxidative stress, and risk of adult brain tumors. *Cancer Epidemiol. Biomarkers. Prev.* 18 1841–1848. 10.1158/1055-9965.epi-09-0197 19505917 PMC2750838

[B12] Blumenthal D. T. Raizer J. J. Rosenblum M. K. Bilsky M. H. Hariharan S. Abrey L. E. (1999). Primary intracranial neoplasms in patients with HIV. *Neurology* 52 1648–1651. 10.1212/WNL.52.8.1648 10331693

[B13] Bochud M. Rousson V. (2010). Usefulness of Mendelian randomization in observational^∗^ epidemiology. *Int. J. Environ. Res. Public Health* 7 711–728. 10.3390/ijerph7030711 20616999 PMC2872313

[B14] Boffetta P. Hashibe M. La Vecchia C. Zatonski W. Rehm J. (2006). The burden of cancer attributable to alcohol drinking. *Int. J. Cancer* 119 884–887. 10.1002/ijc.21903 16557583

[B15] Bondy M. L. Scheurer M. E. Malmer B. Barnholtz-Sloan J. S. Davis F. G. Il’yasova D. (2008). Brain tumor epidemiology: consensus from the brain tumor epidemiology consortium. *Cancer* 113 7(Suppl.) 1953–1968. 10.1002/cncr.23741 18798534 PMC2861559

[B16] Bowden J. Davey Smith G. Burgess S. (2015). Mendelian randomization with invalid instruments: effect estimation and bias detection through Egger regression. *Int. J. Epidemiol.* 44 512–525. 10.1093/ije/dyv080 26050253 PMC4469799

[B17] Braganza M. Z. Kitahara C. M. Berrington de Gonzalez A. Inskip P. D. Johnson K. J. Rajaraman P. (2012). Ionizing radiation and the risk of brain and central nervous system tumors: a systematic review. *Neuro Oncol.* 14 1316–1324. 10.1093/neuonc/nos208 22952197 PMC3480263

[B18] Braganza M. Z. Rajaraman P. Park Y. Inskip P. D. Freedman N. D. Hollenbeck A. R. (2014). Cigarette smoking, alcohol intake, and risk of glioma in the NIH-AARP Diet and Health Study. *Br. J. Cancer* 110 242–248. 10.1038/bjc.2013.611 24335921 PMC3887282

[B19] Brunner C. Davies N. M. Martin R. M. Eeles R. Easton D. Kote-Jarai Z. (2017). Alcohol consumption and prostate cancer incidence and progression: a mendelian randomisation study. *Int. J. Cancer* 140 75–85. 10.1002/ijc.30436 27643404 PMC5111609

[B20] Burgess S. (2014). Sample size and power calculations in mendelian randomization with a single instrumental variable and a binary outcome. *Int. J. Epidemiol.* 43 922–929. 10.1093/ije/dyu005 24608958 PMC4052137

[B21] Burgess S. Dudbridge F. Thompson S. G. (2015). Re: ”Multivariable Mendelian randomization: the use of pleiotropic genetic variants to estimate causal effects”. *Am. J. Epidemiol.* 181 290–291. 10.1093/aje/kwv017 25660081

[B22] Burgess S. Thompson S. G. (2015). Multivariable Mendelian randomization: the use of pleiotropic genetic variants to estimate causal effects. *Am. J. Epidemiol.* 181 251–260. 10.1093/aje/kwu283 25632051 PMC4325677

[B23] Cancer Research United Kingdom (2015). *Brain, Other CNS and Intracranial Tumours Incidence Statistics.* Available: http://www.cancerresearchuk.org/health-professional/cancer-statistics/statistics-by-cancer-type/brain-other-cns-and-intracranial-tumours/incidence#collapseTen [accessed April, 18 2018].

[B24] Cancer Research United Kingdom (2016). *Survival for All Types of Brain Tumour.* Available: http://www.cancerresearchuk.org/health-professional/cancer-statistics/statistics-by-cancer-type/brain-other-cns-and-intracranial-tumours/incidence#collapseTen [accessed 09 2018].

[B25] Carozza S. E. Wrensch M. Miike R. Newman B. Olshan A. F. Savitz D. A. (2000). Occupation and adult Gliomas. *Am. J. Epidemiol.* 152 838–846. 10.1093/aje/152.9.838 11085395

[B26] Castle W. E. (1903). Mendel’s law of heredity. *Science* 18 396–406. 10.1126/science.18.456.396 17752783

[B27] Chen P. Aldape K. Wiencke J. K. Kelsey K. T. Miike R. Davis R. L. (2001). Ethnicity delineates different genetic pathways in malignant Glioma. *Cancer Res.* 61 3949–3954. 11358811

[B28] Cocco P. Dosemeci M. Heineman E. F. (1998). Brain cancer and occupational exposure to lead. *J. Occup. Environ. Med.* 40 937–942. 10.1097/00043764-199811000-00001 9830598

[B29] Corsello S. M. Bittker J. A. Liu Z. Gould J. McCarren P. Hirschman J. E. (2017). The drug repurposing hub: a next-generation drug library and information resource. *Nat. Med.* 23 405–408. 10.1038/nm.4306 28388612 PMC5568558

[B30] Darefsky A. S. Dubrow R. (2009). International variation in the incidence of adult primary malignant neoplasms of the brain and central nervous system. *Cancer Causes Control* 20 1593–1604. 10.1007/s10552-009-9404-1 19633913

[B31] Das A. Tan W. L. Teo J. Smith D. R. (2002). Glioblastoma multiforme in an Asian population: evidence for a distinct genetic pathway. *J. Neurooncol.* 60 117–125. 10.1023/A:1020622415786 12635658

[B32] Daugherty S. E. Moore S. C. Pfeiffer R. M. Inskip P. D. Park Y. Hollenbeck A. (2011). Nonsteroidal anti-inflammatory drugs and glioma in the NIH-AARP diet and health study cohort. *Cancer Prev. Res.* 4 2027–2034. 10.1158/1940-6207.capr-11-0274 21885814 PMC3388115

[B33] Davey Smith G. (2011a). Random allocation in observational data: how small but robust effects could facilitate hypothesis-free causal inference. *Epidemiology* 22 460–463; discussion 467–468. 10.1097/EDE.0b013e31821d0426 21642771

[B34] Davey Smith G. (2011b). Use of genetic markers and gene-diet interactions for interrogating population-level causal influences of diet on health. *Genes Nutr.* 6 27–43. 10.1007/s12263-010-0181-y 21437028 PMC3040803

[B35] Davey Smith G. Ebrahim S. (2003). ‘Mendelian randomization’: can genetic epidemiology contribute to understanding environmental determinants of disease? *Int. J. Epidemiol.* 32 1–22. 10.1093/ije/dyg070 12689998

[B36] Davey Smith G. Hemani G. (2014). Mendelian randomization: genetic anchors for causal inference in epidemiological studies. *Hum. Mol. Genet.* 23 R89–R98. 10.1093/hmg/ddu328 25064373 PMC4170722

[B37] Davey Smith G. Paternoster L. Relton C. (2017). When will mendelian randomization become relevant for clinical practice and public health? *JAMA* 317 589–591. 10.1001/jama.2016.21189 28196238

[B38] Davis F. G. McCarthy B. Jukich P. (1999). The descriptive epidemiology of brain tumors. *Neuroimaging Clin. N. Am.* 9 581–594. 10517935

[B39] Didelez V. Sheehan N. (2007). Mendelian randomization as an instrumental variable approach to causal inference. *Stat. Methods Med. Res.* 16 309–330. 10.1177/0962280206077743 17715159

[B40] Disney-Hogg L. Cornish A. J. Sud A. Law P. J. Kinnersley B. Jacobs D. I. (2018a). Impact of atopy on risk of glioma: a Mendelian randomisation study. *BMC Med.* 16:42. 10.1186/s12916-018-1027-5 29540232 PMC5853158

[B41] Disney-Hogg L. Sud A. Law P. J. Cornish A. J. Kinnersley B. Ostrom Q. T. (2018b). Influence of obesity-related risk factors in the aetiology of Glioma. *Br. J. Cancer* 118 1020–1027. 10.1038/s41416-018-0009-x 29531326 PMC5931112

[B42] Dubrow R. Darefsky A. S. (2011). Demographic variation in incidence of adult glioma by subtype, United States, 1992-2007. *BMC Cancer* 11:325. 10.1186/1471-2407-11-325 21801393 PMC3163630

[B43] Esteller M. Garcia-Foncillas J. Andion E. Goodman S. N. Hidalgo O. F. Vanaclocha V. (2000). Inactivation of the DNA-repair gene MGMT and the clinical response of gliomas to alkylating agents. *N. Engl. J. Med.* 343 1350–1354. 10.1056/nejm200011093431901 11070098

[B44] Evans D. M. Brion M. J. Paternoster L. Kemp J. P. McMahon G. Munafo M. (2013). Mining the human phenome using allelic scores that index biological intermediates. *PLoS Genet.* 9:e1003919. 10.1371/journal.pgen.1003919 24204319 PMC3814299

[B45] Evans D. M. Davey Smith G. (2015). Mendelian randomization: new applications in the coming age of hypothesis-free causality. *Annu. Rev. Genomics Hum. Genet.* 16 327–350. 10.1146/annurev-genom-090314-050016 25939054

[B46] Evans D. M. Smith G. D. (2015). Mendelian randomization: new applications in the coming age of hypothesis-free causality. *Annu. Rev. Genomics Hum. Genet.* 16 327–350. 10.1146/annurev-genom-090314-050016 25939054

[B47] Ference B. A. Ginsberg H. N. Graham I. Ray K. K. Packard C. J. Bruckert E. (2017). Low-density lipoproteins cause atherosclerotic cardiovascular disease. 1. Evidence from genetic, epidemiologic, and clinical studies. A consensus statement from the European Atherosclerosis Society Consensus Panel. *Eur. Heart J.* 38 2459–2472. 10.1093/eurheartj/ehx144 28444290 PMC5837225

[B48] Flegal K. M. Graubard B. I. Williamson D. F. Cooper R. S. (2011). Reverse causation and illness-related weight loss in observational studies of body weight and mortality. *Am. J. Epidemiol.* 173 1–9. 10.1093/aje/kwq341 21059807

[B49] Frei P. Poulsen A. H. Johansen C. Olsen J. H. Steding-Jessen M. Schüz J. (2011). Use of mobile phones and risk of brain tumours: update of Danish cohort study. *BMJ* 343:3. 10.1136/bmj.d6387 22016439 PMC3197791

[B50] Gaist D. García-Rodríguez L. A. Sørensen H. T. Hallas J. Friis S. (2013). Use of low-dose aspirin and non-aspirin nonsteroidal anti-inflammatory drugs and risk of Glioma: a case–control study. *Br. J. Cancer* 108 1189–1194. 10.1038/bjc.2013.87 23449355 PMC3619088

[B51] Gehring K. Sitskoorn M. M. Gundy C. M. Sikkes S. A. Klein M. Postma T. J. (2009). Cognitive rehabilitation in patients with gliomas: a randomized, controlled trial. *J. Clin. Oncol.* 27 3712–3722. 10.1200/jco.2008.20.5765 19470928

[B52] Giles G. G. McNeil J. J. Donnan G. Webley C. Staples M. P. Ireland P. D. (1994). Dietary factors and the risk of glioma in adults: results of a case-control study in Melbourne. Australia. *Int. J. Cancer* 59 357–362. 10.1002/ijc.2910590311 7927941

[B53] Glynn R. J. (2010). Promises and limitations of mendelian randomization for evaluation of biomarkers. *Clin. Chem.* 56 388–390. 10.1373/clinchem.2009.142513 20093553

[B54] Greenland S. (2000). An introduction to instrumental variables for epidemiologists. *Int. J. Epidemiol.* 29 722–729. 10.1093/ije/29.4.722 10922351

[B55] Hall J. R. Short S. C. (2009). Management of glioblastoma multiforme in HIV patients: a case series and review of published studies. *Clin. Oncol.* 21 591–597. 10.1016/j.clon.2009.04.006 19589665

[B56] Hammer G. P. du Prel J. B. Blettner M. (2009). Avoiding bias in observational studies: part 8 in a series of articles on evaluation of scientific publications. *Dtsch Arztebl. Int.* 106 664–668. 10.3238/arztebl.2009.0664 19946431 PMC2780010

[B57] Hanahan D. Weinberg R. A. (2011). Hallmarks of cancer: the next generation. *Cell* 144 646–674. 10.1016/j.cell.2011.02.013 21376230

[B58] Hart C. L. Davey Smith G. Gruer L. Watt G. C. (2010). The combined effect of smoking tobacco and drinking alcohol on cause-specific mortality: a 30 year cohort study. *BMC Public Health* 10:789. 10.1186/1471-2458-10-789 21184680 PMC3022858

[B59] Hartwig F. P. Davies N. M. Hemani G. Davey Smith G. (2016). Two-sample Mendelian randomization: avoiding the downsides of a powerful, widely applicable but potentially fallible technique. *Int. J. Epidemiol.* 45 1717–1726. 10.1093/ije/dyx028 28338968 PMC5722032

[B60] Haycock P. C. Burgess S. Nounu A. Zheng J. Okoli G. N. Bowden J. (2017). Association between telomere length and risk of cancer and non-neoplastic diseases: a mendelian randomization study. *JAMA Oncol.* 3 636–651. 10.1001/jamaoncol.2016.5945 28241208 PMC5638008

[B61] Haycock P. C. Burgess S. Wade K. H. Bowden J. Relton C. Davey Smith G. (2016). Best (but oft-forgotten) practices: the design, analysis, and interpretation of Mendelian randomization studies. *Am. J. Clin. Nutr.* 103 965–978. 10.3945/ajcn.115.118216 26961927 PMC4807699

[B62] Hemani G. Zheng J. Elsworth B. Wade K. H. Haberland V. Baird D. (2018). The MR-Base platform supports systematic causal inference across the human phenome. *eLife* 7:e34408. 10.7554/eLife.34408 29846171 PMC5976434

[B63] Hemani G. Zheng J. Wade K. H. Laurin C. Elsworth B. Burgess S. (2016). MR-Base: a platform for systematic causal inference across the phenome using billions of genetic associations. *bioRxiv* [Preprint] 10.1101/078972

[B64] Holick C. N. Smith S. G. Giovannucci E. Michaud D. S. (2010). Coffee, tea, caffeine intake, and risk of adult glioma in three prospective cohort studies. *Cancer Epidemiol. Biomark. Prev.* 19 39–47. 10.1158/1055-9965.Epi-09-0732 20056621 PMC2943732

[B65] Houben M. P. Louwman W. J. Tijssen C. C. Teepen J. L. van Duijn C. M. Coebergh J. W. (2004). Hypertension as a risk factor for glioma? Evidence from a population-based study of comorbidity in glioma patients. *Ann. Oncol.* 15 1256–1260. 10.1093/annonc/mdh306 15277267

[B66] Inoue A. Solon G. (2010). Two-sample instrumental variables estimators. *Rev. Econ. Stat.* 92 557–561. 10.1162/REST_a_00011 27667880

[B67] Iturrieta-Zuazo I. Walter S. (2015). Mendelian randomization: present and future of epidemiological studies in cardiology. *Revista Española Cardiología* 68 87–91. 10.1016/j.recesp.2014.06.026 25449812

[B68] Jukich P. J. McCarthy B. J. Surawicz T. S. Freels S. Davis F. G. (2001). Trends in incidence of primary brain tumors in the United States, 1985-1994. *Neuro Oncol.* 3 141–151. 10.1093/neuonc/3.3.141 11465394 PMC1920618

[B69] Kabat G. C. Park Y. Hollenbeck A. R. Schatzkin A. Rohan T. E. (2011). Reproductive factors and exogenous hormone use and risk of adult glioma in women in the NIH-AARP diet and health study. *Int. J. Cancer* 128 944–950. 10.1002/ijc.25413 20473903 PMC3491883

[B70] Kaplan S. Novikov I. Modan B. (1997). Nutritional factors in the etiology of brain tumors: potential role of nitrosamines, fat, and cholesterol. *Am. J. Epidemiol.* 146 832–841. 10.1093/oxfordjournals.aje.a009201 9384204

[B71] Kelly P. J. (2010). Gliomas: survival, origin and early detection. *Surg. Neurol. Int.* 1:96. 10.4103/2152-7806.74243 21246059 PMC3019361

[B72] Kemp J. P. Sayers A. Smith G. D. Tobias J. H. Evans D. M. (2016). Using Mendelian randomization to investigate a possible causal relationship between adiposity and increased bone mineral density at different skeletal sites in children. *Int. J. Epidemiol.* 45 1560–1572. 10.1093/ije/dyw079 27215616 PMC5100609

[B73] Khuder S. A. Mutgi A. B. Schaub E. A. (1998). Meta-analyses of brain cancer and farming. *Am. J. Ind. Med.* 34 252–260. 10.1002/(SICI)1097-0274(199809)34:3<252::AID-AJIM7>3.0.CO;2-X 9698994

[B74] Kim Y. I. (2003). Role of folate in colon cancer development and progression. *J. Nutr.* 133(11 Suppl. 1) 3731s–3739s. 10.1093/jn/133.11.3731S 14608107

[B75] Kinnersley B. Houlston R. S. Bondy M. L. (2018). Genome-wide association studies in Glioma. *Cancer Epidemiol. Biomark. Prev.* 27 418–428. 10.1158/1055-9965.epi-17-1080 29382702 PMC5931394

[B76] Kitahara C. M. Linet M. S. Brenner A. V. Wang S. S. Melin B. S. Wang Z. (2014). Personal history of diabetes, genetic susceptibility to diabetes, and risk of brain glioma: a pooled analysis of observational studies. *Cancer Epidemiol. Biomarkers. Prev.* 23 47–54. 10.1158/1055-9965.epi-13-0913 24220915 PMC3947107

[B77] Krishnan G. Felini M. Carozza S. E. Miike R. Chew T. Wrensch M. (2003). Occupation and adult Gliomas in the San Francisco Bay Area. *J. Occup. Environ. Med.* 45 639–647. 10.1097/01.jom.0000069245.06498.48 12802217

[B78] Kuratsu J. Takeshima H. Ushio Y. (2001). Trends in the incidence of primary intracranial tumors in Kumamoto, Japan. *Int. J. Clin. Oncol.* 6 183–191. 10.1007/pl00023928 11706556

[B79] Kyritsis A. P. Bondy M. L. Levin V. A. (2011). Modulation of glioma risk and progression by dietary nutrients and antiinflammatory agents. *Nutr. Cancer* 63 174–184. 10.1080/01635581.2011.523807 21302177 PMC3047463

[B80] Lam T. V. Agovino P. Niu X. Roche L. (2007). Linkage study of cancer risk among lead-exposed workers in New Jersey. *Sci. Total Environ.* 372 455–462. 10.1016/j.scitotenv.2006.10.018 17129599

[B81] Larsson S. C. Traylor M. Malik R. Dichgans M. Burgess S. Markus H. S. (2017). Modifiable pathways in Alzheimer’s disease: mendelian randomisation analysis. *BMJ* 359:j5375. 10.1136/bmj.j5375 29212772 PMC5717765

[B82] Lawlor D. A. Davey Smith G. Bruckdorfer K. R. Kundu D. Ebrahim S. (2004). Observational versus randomised trial evidence. *Lancet* 364 755–756. 10.1016/s0140-6736(04)16926-2 15337394

[B83] Lawlor D. A. Harbord R. M. Sterne J. A. Timpson N. Davey Smith G. (2008). Mendelian randomization: using genes as instruments for making causal inferences in epidemiology. *Stat. Med.* 27 1133–1163. 10.1002/sim.3034 17886233

[B84] Lee S. T. Bracci P. Zhou M. Rice T. Wiencke J. Wrensch M. (2014). Interaction of allergy history and antibodies to specific varicella-zoster virus proteins on glioma risk. *Int. J. Cancer* 134 2199–2210. 10.1002/ijc.28535 24127236 PMC3951480

[B85] Li H. X. Meng H. Y. Peng X. X. Zong Q. Zhang K. Han G. L. (2015). A meta-analysis of association between pesticides exposure and glioma risk in adults. *J. Craniofac. Surg.* 26 e672–e673. 10.1097/scs.0000000000001707 26439196

[B86] Liao L. M. Friesen M. C. Xiang Y. B. Cai H. Koh D. H. Ji B. T. (2016). Occupational lead exposure and associations with selected cancers: the shanghai men’s and women’s health study cohorts. *Environ. Health Perspect.* 124 97–103. 10.1289/ehp.1408171 26091556 PMC4710592

[B87] Linos E. Raine T. Alonso A. Michaud D. (2007). Atopy and risk of brain tumors: a meta-analysis. *J. Natl. Cancer Inst.* 99 1544–1550. 10.1093/jnci/djm170 17925535

[B88] Little R. B. Madden M. H. Thompson R. C. Olson J. J. Larocca R. V. Pan E. (2013). Anthropometric factors in relation to risk of glioma. *Cancer Causes Control* 24 1025–1031. 10.1007/s10552-013-0178-0 23456313 PMC3633685

[B89] Louis D. N. Perry A. Reifenberger G. von Deimling A. Figarella-Branger D. Cavenee W. K. (2016). The 2016 world health organization classification of tumors of the central nervous system: a summary. *Acta Neuropathol.* 131 803–820. 10.1007/s00401-016-1545-1 27157931

[B90] Low K. B. (2001). “Pleiotropy A2 - brenner, sydney,” in *Encyclopedia of Genetics* ed. Miller J. H. (New York, NY: Academic Press) 1490–1491.

[B91] Malerba S. Galeone C. Pelucchi C. Turati F. Hashibe M. La Vecchia C. (2013). A meta-analysis of coffee and tea consumption and the risk of glioma in adults. *Cancer Causes Control* 24 267–276. 10.1007/s10552-012-0126-4 23247638

[B92] McLendon R. E. Robinson J. S. Jr. Chambers D. B. Grufferman S. Burger P. C. (1985). The glioblastoma multiforme in georgia, 1977-1981. *Cancer* 56 894–897. 10.1002/1097-0142(19850815)56:4<894::AID-CNCR2820560432>3.0.CO;2-# 2990658

[B93] Melin B. S. Barnholtz-Sloan J. S. Wrensch M. R. Johansen C. Il’yasova D. Kinnersley B. (2017). Genome-wide association study of glioma subtypes identifies specific differences in genetic susceptibility to glioblastoma and non-glioblastoma tumors. *Nat. Genet.* 49 789–794. 10.1038/ng.3823 28346443 PMC5558246

[B94] Meyer R. M. (2010). Generalizing the results of cancer clinical trials. *J. Clin. Oncol.* 28 187–189. 10.1200/jco.2009.25.8608 19933900

[B95] Michaud D. S. Holick C. N. Batchelor T. T. Giovannucci E. Hunter D. J. (2009). Prospective study of meat intake and dietary nitrates, nitrites, and nitrosamines and risk of adult glioma. *Am. J. Clin. Nutr.* 90 570–577. 10.3945/ajcn.2008.27199 19587083 PMC2728643

[B96] Mochizuki S. Iwadate Y. Namba H. Yoshida Y. Yamaura A. Sakiyama S. (1999). Homozygous deletion of the p16/MTS-1/CDKN2 gene in malignant gliomas is infrequent among Japanese patients. *Int. J. Oncol.* 15 983–989. 10.3892/ijo.15.5.983 10536183

[B97] Mokry L. E. Ahmad O. Forgetta V. Thanassoulis G. Richards J. B. (2015). Mendelian randomisation applied to drug development in cardiovascular disease: a review. *J. Med. Genet.* 52 71–79. 10.1136/jmedgenet-2014-102438 25515070

[B98] Montgomery A. A. Peters T. J. Little P. (2003). Design, analysis and presentation of factorial randomised controlled trials. *BMC Med. Res. Methodol.* 3:26. 10.1186/1471-2288-3-26 14633287 PMC305359

[B99] Navas-Acien A. Pollan M. Gustavsson P. Plato N. (2002). Occupation, exposure to chemicals and risk of gliomas and meningiomas in Sweden. *Am. J. Ind. Med.* 42 214–227. 10.1002/ajim.10107 12210690

[B100] Neglia J. P. Robison L. L. Stovall M. Liu Y. Packer R. J. Hammond S. (2006). New primary neoplasms of the central nervous system in survivors of childhood cancer: a report from the childhood cancer survivor study. *J. Natl. Cancer Ins.* 98 1528–1537. 10.1093/jnci/djj411 17077355

[B101] Nelson M. R. Tipney H. Painter J. L. Shen J. Nicoletti P. Shen Y. (2015). The support of human genetic evidence for approved drug indications. *Nat. Genet.* 47 856–860. 10.1038/ng.3314 26121088

[B102] Olson R. A. Brastianos P. K. Palma D. A. (2011). Prognostic and predictive value of epigenetic silencing of MGMT in patients with high grade gliomas: a systematic review and meta-analysis. *J. Neurooncol.* 105 325–335. 10.1007/s11060-011-0594-5 21523485

[B103] Ostrom Q. T. Bauchet L. Davis F. G. Deltour I. Fisher J. L. Langer C. E. (2014). The epidemiology of glioma in adults: a ”state of the science” review. *Neuro Oncol.* 16 896–913. 10.1093/neuonc/nou087 24842956 PMC4057143

[B104] Ostrom Q. T. Gittleman H. Farah P. Ondracek A. Chen Y. Wolinsky Y. (2013). CBTRUS statistical report: primary brain and central nervous system tumors diagnosed in the United States in 2006-2010. *Neuro Oncol.* 15(Suppl. 2) ii1–ii56. 10.1093/neuonc/not151 24137015 PMC3798196

[B105] Parent M. E. Turner M. C. Lavoue J. Richard H. Figuerola J. Kincl L. (2017). Lifetime occupational exposure to metals and welding fumes, and risk of glioma: a 7-country population-based case-control study. *Environ. Health* 16:90. 10.1186/s12940-017-0300-y 28841833 PMC5574088

[B106] Paternoster L. Tilling K. Davey Smith G. (2017). Genetic epidemiology and Mendelian randomization for informing disease therapeutics: conceptual and methodological challenges. *PLoS Genet.* 13:e1006944. 10.1371/journal.pgen.1006944 28981501 PMC5628782

[B107] Preston-Martin S. Mack W. (1991). Gliomas and meningiomas in men in Los Angeles county: investigation of exposures to N-nitroso compounds. *IARC Sci. Publ.* 105 197–203. 1855850

[B108] Pukkala E. Martinsen J. I. Lynge E. Gunnarsdottir H. K. Sparén P. Tryggvadottir L. (2009). Occupation and cancer – follow-up of 15 million people in five Nordic countries. *Acta Oncol.* 48 646–790. 10.1080/02841860902913546 19925375

[B109] Qi L. (2009). Mendelian randomization in nutritional epidemiology. *Nutr. Rev.* 67 439–450. 10.1111/j.1753-4887.2009.00218.x 19674341 PMC3671930

[B110] Qi Z. Y. Shao C. Yang C. Wang Z. Hui G. Z. (2014). Alcohol consumption and risk of glioma: a meta-analysis of 19 observational studies. *Nutrients* 6 504–516. 10.3390/nu6020504 24473233 PMC3942713

[B111] Quach P. El Sherif R. Gomes J. Krewksi D. (2017). A systematic review of the risk factors associated with the onset and progression of primary brain tumours. *Neurotoxicology* 61 214–232. 10.1016/j.neuro.2016.05.009 27212451

[B112] Rajaraman P. Stewart P. A. Samet J. M. Schwartz B. S. Linet M. S. Zahm S. H. (2006). Lead, genetic susceptibility, and risk of adult brain tumors. *Cancer Epidemiol. Biomark. Prev.* 15 2514–2520. 10.1158/1055-9965.epi-06-0482 17164378

[B113] Reardon D. A. Wen P. Y. (2006). Therapeutic advances in the treatment of glioblastoma: rationale and potential role of targeted agents. *Oncologist* 11 152–164. 10.1634/theoncologist.11-2-152 16476836

[B114] Reifenberger G. Hentschel B. Felsberg J. Schackert G. Simon M. Schnell O. (2012). Predictive impact of MGMT promoter methylation in glioblastoma of the elderly. *Int. J. Cancer* 131 1342–1350. 10.1002/ijc.27385 22139906

[B115] Relton C. L. Davey Smith G. (2012). Two-step epigenetic Mendelian randomization: a strategy for establishing the causal role of epigenetic processes in pathways to disease. *Int. J. Epidemiol.* 41 161–176. 10.1093/ije/dyr233 22422451 PMC3304531

[B116] Rice T. Lachance D. H. Molinaro A. M. Eckel-Passow J. E. Walsh K. M. Barnholtz-Sloan J. (2016). Understanding inherited genetic risk of adult glioma – a review. *Neuro Oncol. Pract.* 3 10–16. 10.1093/nop/npv026 26941959 PMC4774334

[B117] Robertson J. T. Gunter B. C. Somes G. W. (2002). Racial differences in the incidence of gliomas: a retrospective study from Memphis, Tennessee. *Br. J. Neurosurg.* 16 562–566. 10.1080/02688690209168361 12617237

[B118] Sadetzki S. Chetrit A. Freedman L. Stovall M. Modan B. Novikov I. (2005). Long-term follow-up for brain tumor development after childhood exposure to ionizing radiation for tinea capitis. *Radiat. Res.* 163 424–432. 10.1667/RR3329 15799699

[B119] Safaeian M. Rajaraman P. Hartge P. Yeager M. Linet M. Butler M. A. (2013). Joint effects between five identified risk variants, allergy, and autoimmune conditions on glioma risk. *Cancer Causes Control* 24 1885–1891. 10.1007/s10552-013-0244-7 23903690 PMC4074857

[B120] Samkange-Zeeb F. Schlehofer B. Schuz J. Schlaefer K. Berg-Beckhoff G. Wahrendorf J. (2010). Occupation and risk of glioma, meningioma and acoustic neuroma: results from a German case-control study (interphone study group, Germany). *Cancer Epidemiol.* 34 55–61. 10.1016/j.canep.2009.12.003 20061201

[B121] Sayon-Orea C. Martinez-Gonzalez M. A. Bes-Rastrollo M. (2011). Alcohol consumption and body weight: a systematic review. *Nutr. Rev.* 69 419–431. 10.1111/j.1753-4887.2011.00403.x 21790610

[B122] Scheurer M. E. Amirian E. S. Davlin S. L. Rice T. Wrensch M. Bondy M. L. (2011). Effects of antihistamine and anti-inflammatory medication use on risk of specific glioma histologies. *Int. J. Cancer* 129 2290–2296. 10.1002/ijc.25883 21190193 PMC3125483

[B123] Schoemaker M. J. Swerdlow A. J. Hepworth S. J. McKinney P. A. van Tongeren M. Muir K. R. (2006). History of allergies and risk of glioma in adults. *Int. J. Cancer* 119 2165–2172. 10.1002/ijc.22091 16823851

[B124] Schüz J. Jacobsen R. Olsen J. H. Boice J. J. D. McLaughlin J. K. Johansen C. (2006). Cellular telephone use and cancer risk: update of a nationwide danish cohort. *J. Natl. Cancer Inst.* 98 1707–1713. 10.1093/jnci/djj464 17148772

[B125] Schwartzbaum J. Ahlbom A. Malmer B. Lonn S. Brookes A. J. Doss H. (2005). Polymorphisms associated with asthma are inversely related to glioblastoma multiforme. *Cancer Res.* 65 6459–6465. 10.1158/0008-5472.can-04-3728 16024651 PMC1762912

[B126] Schwartzbaum J. Edlinger M. Zigmont V. Stattin P. Rempala G. A. Nagel G. (2017). Associations between prediagnostic blood glucose levels, diabetes, and glioma. *Sci. Rep.* 7:1436. 10.1038/s41598-017-01553-2 28469238 PMC5431098

[B127] Seliger C. Meier C. R. Becker C. Jick S. S. Bogdahn U. Hau P. (2016a). Statin use and risk of glioma: population-based case-control analysis. *Eur. J. Epidemiol.* 31 947–952. 10.1007/s10654-016-0145-7 27041698

[B128] Seliger C. Ricci C. Meier C. R. Bodmer M. Jick S. S. Bogdahn U. (2016b). Diabetes, use of antidiabetic drugs, and the risk of glioma. *Neuro Oncol.* 18 340–349. 10.1093/neuonc/nov100 26093337 PMC4767232

[B129] Sergentanis T. N. Tsivgoulis G. Perlepe C. Ntanasis-Stathopoulos I. Tzanninis I. G. Sergentanis I. N. (2015). Obesity and risk for brain/CNS tumors, gliomas and meningiomas: a meta-analysis. *PLoS One* 10:e0136974. 10.1371/journal.pone.0136974 26332834 PMC4558052

[B130] Shao C. Zhao W. Qi Z. He J. (2016). Smoking and glioma risk: evidence from a meta-analysis of 25 observational studies. *Medicine* 95:e2447. 10.1097/md.0000000000002447 26765433 PMC4718259

[B131] Sheehan N. A. Didelez V. Burton P. R. Tobin M. D. (2008). Mendelian randomisation and causal inference in observational epidemiology. *PLoS Med.* 5:e177. 10.1371/journal.pmed.0050177 18752343 PMC2522255

[B132] Shi J. Marconett C. N. Duan J. Hyland P. L. Li P. Wang Z. (2014). Characterizing the genetic basis of methylome diversity in histologically normal human lung tissue. *Nat. Commun.* 5:3365. 10.1038/ncomms4365 24572595 PMC3982882

[B133] Smith G. D. (2010). Mendelian randomization for strengthening causal inference in observational studies:application to gene × environment interactions. *Perspect. Psychol. Sci.* 5 527–545. 10.1177/1745691610383505 26162196

[B134] Smith G. D. Ebrahim S. (2004). Mendelian randomization: prospects, potentials, and limitations. *Int. J. Epidemiol.* 33 30–42. 10.1093/ije/dyh132 15075143

[B135] Sofat R. Hingorani A. D. Smeeth L. Humphries S. E. Talmud P. J. Cooper J. (2010). Separating the mechanism-based and off-target actions of cholesteryl ester transfer protein inhibitors with CETP gene polymorphisms. *Circulation* 121 52–62. 10.1161/circulationaha.109.865444 20026784 PMC2811869

[B136] Stupp R. Hegi M. E. Mason W. P. van den Bent M. J. Taphoorn M. J. Janzer R. C. (2009). Effects of radiotherapy with concomitant and adjuvant temozolomide versus radiotherapy alone on survival in glioblastoma in a randomised phase III study: 5-year analysis of the EORTC-NCIC trial. *Lancet Oncol.* 10 459–466. 10.1016/s1470-2045(09)70025-7 19269895

[B137] Sun B. B. Maranville J. C. Peters J. E. Stacey D. Staley J. R. Blackshaw J. (2018). Genomic atlas of the human plasma proteome. *Nature* 558 73–79. 10.1038/s41586-018-0175-2 29875488 PMC6697541

[B138] Sunderman F. W. Jr. (2001). Nasal toxicity, carcinogenicity, and olfactory uptake of metals. *Ann. Clin. Lab. Sci.* 31 3–24. 11314863

[B139] Takahashi H. Cornish A. J. Sud A. Law P. J. Kinnersley B. Ostrom Q. T. (2018). Mendelian randomisation study of the relationship between vitamin D and risk of glioma. *Sci. Rep.* 8:2339. 10.1038/s41598-018-20844-w 29402980 PMC5799201

[B140] Tamimi A. F. Juweid M. (2017). “Epidemiology and outcome of glioblastoma,” in *Glioblastoma* ed. De Vleeschouwer S. (Brisbane, QLD: Codon Publications Copyright). 29251870

[B141] Taylor A. J. Little M. P. Winter D. L. Sugden E. Ellison D. W. Stiller C. A. (2010). Population-based risks of CNS tumors in survivors of childhood cancer: the British childhood cancer survivor study. *J. Clin. Oncol.* 28 5287–5293. 10.1200/jco.2009.27.0090 21079138 PMC4809645

[B142] Testino G. (2011). The burden of cancer attributable to alcohol consumption. *Maedica* 6 313–320. 22879847 PMC3391950

[B143] Timpson N. J. Nordestgaard B. G. Harbord R. M. Zacho J. Frayling T. M. Tybjærg-Hansen A. (2010). C-reactive protein levels and body mass index: elucidating direction of causation through reciprocal Mendelian randomization. *Int. J. Obes.* 35 300. 10.1038/ijo.2010.137 20714329 PMC4783860

[B144] Touat M. Idbaih A. Sanson M. Ligon K. L. (2017). Glioblastoma targeted therapy: updated approaches from recent biological insights. *Ann. Oncol.* 28 1457–1472. 10.1093/annonc/mdx106 28863449 PMC5834086

[B145] U.S.National Library of Medicine (2018). *Intraoperative Ultrasound Guided Glioma Surgery; a Randomised, Controlled Trial.* Available at: https://clinicaltrials.gov/ct2/show/NCT03531333?id=NCT03531333&rank=1&load=cart

[B146] Urbanska K. Sokolowska J. Szmidt M. Sysa P. (2014). Glioblastoma multiforme - an overview. *Contemp. Oncol.* 18 307–312. 10.5114/wo.2014.40559 25477751 PMC4248049

[B147] van Wijngaarden E. Dosemeci M. (2006). Brain cancer mortality and potential occupational exposure to lead: findings from the national longitudinal mortality study, 1979-1989. *Int. J. Cancer* 119 1136–1144. 10.1002/ijc.21947 16570286

[B148] VanderWeele T. J. Tchetgen Tchetgen E. J. Cornelis M. Kraft P. (2014). Methodological challenges in mendelian randomization. *Epidemiology* 25 427–435. 10.1097/ede.0000000000000081 24681576 PMC3981897

[B149] Visser O. Ardanaz E. Botta L. Sant M. Tavilla A. Minicozzi P. (2015). Survival of adults with primary malignant brain tumours in Europe; results of the EUROCARE-5 study. *Eur. J. Cancer* 51 2231–2241. 10.1016/j.ejca.2015.07.032 26421825

[B150] Vuorinen V. Hinkka S. Farkkila M. Jaaskelainen J. (2003). Debulking or biopsy of malignant glioma in elderly people - a randomised study. *Acta Neurochir.* 145 5–10. 10.1007/s00701-002-1030-6 12545256

[B151] Wald A. (1940). The fitting of straight lines if both variables are subject to error. *Ann. Math. Stat.* 11 284–300. 10.1214/aoms/1177731868

[B152] Walsh K. M. Codd V. Rice T. Nelson C. P. Smirnov I. V. McCoy L. S. (2015). Longer genotypically-estimated leukocyte telomere length is associated with increased adult glioma risk. *Oncotarget* 6 42468–42477. 10.18632/oncotarget.6468 26646793 PMC4767445

[B153] Walsh K. M. Codd V. Smirnov I. V. Rice T. Decker P. A. Hansen H. M. (2014). Variants near TERT and TERC influencing telomere length are associated with high-grade glioma risk. *Nat. Genet.* 46 731–735. 10.1038/ng.3004 24908248 PMC4074274

[B154] Wang B. Du Y. (2013). Cadmium and its neurotoxic effects. *Oxid. Med. Cell. Longev.* 2013:898034. 10.1155/2013/898034 23997854 PMC3753751

[B155] Wang G. Xu S. Cao C. Dong J. Chu Y. He G. (2016). Evidence from a large-scale meta-analysis indicates eczema reduces the incidence of glioma. *Oncotarget* 7 62598–62606. 10.18632/oncotarget.11545 27566584 PMC5308749

[B156] Wang H. Xu T. Jiang Y. Xu H. Yan Y. Fu D. (2015). The challenges and the promise of molecular targeted therapy in malignant gliomas. *Neoplasia* 17 239–255. 10.1016/j.neo.2015.02.002 25810009 PMC4372648

[B157] Wen P. Y. Kesari S. (2008). Malignant gliomas in adults. *N. Engl. J. Med.* 359 492–507. 10.1056/NEJMra0708126 18669428

[B158] Wick W. Platten M. Meisner C. Felsberg J. Tabatabai G. Simon M. (2012). Temozolomide chemotherapy alone versus radiotherapy alone for malignant astrocytoma in the elderly: the NOA-08 randomised, phase 3 trial. *Lancet Oncol.* 13 707–715. 10.1016/s1470-2045(12)70164-x 22578793

[B159] Wiedmann M. K. H. Brunborg C. Di Ieva A. Lindemann K. Johannesen T. B. Vatten L. (2017). The impact of body mass index and height on the risk for glioblastoma and other glioma subgroups: a large prospective cohort study. *Neuro Oncol.* 19 976–985. 10.1093/neuonc/now272 28040713 PMC5570185

[B160] Wigertz A. Lonn S. Schwartzbaum J. Hall P. Auvinen A. Christensen H. C. (2007). Allergic conditions and brain tumor risk. *Am. J. Epidemiol.* 166 941–950. 10.1093/aje/kwm203 17646205

[B161] Wurtz P. Kangas A. J. Soininen P. Lehtimaki T. Kahonen M. Viikari J. S. (2013). Lipoprotein subclass profiling reveals pleiotropy in the genetic variants of lipid risk factors for coronary heart disease: a note on Mendelian randomization studies. *J. Am. Coll. Cardiol.* 62 1906–1908. 10.1016/j.jacc.2013.07.085 24055740

[B162] Yang M. Guo W. Yang C. Tang J. Huang Q. Feng S. (2017). Mobile phone use and glioma risk: a systematic review and meta-analysis. *PLoS One* 12:e0175136. 10.1371/journal.pone.0175136 28472042 PMC5417432

[B163] Yarmolinsky J. Wade K. H. Richmond R. C. Langdon R. J. Bull C. J. Tilling K. M. (2017). Causal inference in cancer epidemiology: what is the role of Mendelian randomization? *bioRxiv* [Preprint] 10.1101/223966 PMC6522350 29941659

[B164] Zhao H. Cai W. Su S. Zhi D. Lu J. Liu S. (2014). Allergic conditions reduce the risk of glioma: a meta-analysis based on 128,936 subjects. *Tumour Biol.* 35 3875–3880. 10.1007/s13277-013-1514-4 24347487

[B165] Zhao L. Zheng Z. Huang P. (2016). Diabetes mellitus and the risk of glioma: a meta-analysis. *Oncotarget* 7 4483–4489. 10.18632/oncotarget.6605 26683358 PMC4826220

[B166] Zheng J. Baird D. Borges M.-C. Bowden J. Hemani G. Haycock P. (2017). Recent developments in mendelian randomization studies. *Curr. Epidemiol. Rep.* 4 330–345. 10.1007/s40471-017-0128-6 29226067 PMC5711966

[B167] Zheng T. Cantor K. P. Zhang Y. Keim S. Lynch C. F. (2001). Occupational risk factors for brain cancer: a population-based case-control study in Iowa. *J. Occup. Environ. Med.* 43 317–324. 10.1097/00043764-200104000-00005 11322092

[B168] Zhou S. Wang X. Tan Y. Qiu L. Fang H. Li W. (2015). Association between vitamin C intake and glioma risk: evidence from a meta-analysis. *Neuroepidemiology* 44 39–44. 10.1159/000369814 25720916

